# A Biased Bayesian Inference for Decision-Making and Cognitive Control

**DOI:** 10.3389/fnins.2018.00734

**Published:** 2018-10-12

**Authors:** Kaosu Matsumori, Yasuharu Koike, Kenji Matsumoto

**Affiliations:** ^1^Tamagawa University Brain Science Institute, Machida, Tokyo, Japan; ^2^Department of Information Processing, Tokyo Institute of Technology, Yokohama, Kanagawa, Japan; ^3^Institute of Innovative Research, Tokyo Institute of Technology, Yokohama, Kanagawa, Japan

**Keywords:** sub-optimality, parameter estimation, probability judgment, probabilistic population codes, gain modulation, two-alternative forced choice, cognitive control, computational psychiatry

## Abstract

Although classical decision-making studies have assumed that subjects behave in a Bayes-optimal way, the sub-optimality that causes biases in decision-making is currently under debate. Here, we propose a synthesis based on exponentially-biased Bayesian inference, including various decision-making and probability judgments with different bias levels. We arrange three major parameter estimation methods in a two-dimensional bias parameter space (prior and likelihood), of the biased Bayesian inference. Then, we discuss a neural implementation of the biased Bayesian inference on the basis of changes in weights in neural connections, which we regarded as a combination of leaky/unstable neural integrator and probabilistic population coding. Finally, we discuss mechanisms of cognitive control which may regulate the bias levels.

## Introduction

Decision-making and cognitive control, which are well developed in primates (especially humans), are important for adaptive behaviors in a changing environment with uncertainty (Fuster, 2015). Many studies in various research fields such as Psychology, Neuroscience, and Engineering have suggested that decision-making and cognitive control are implemented differently in brain regions which contribute to processing these functions (Stuss and Knight, 2013; Fuster, 2015). However, the computational principles underlying these processes are still under debate (Gazzaniga, 2009; Friston, 2010).

Recent theoretical studies suggest a unified view accounting for various cognitive functions including decision-making and cognitive control on the basis of Bayesian inference (which forces us to re-assess our prior subjective beliefs using currently observed data, while considering uncertainty as well), as means of obtaining the distributions (Friston, 2010; Beck et al., 2012; Pouget et al., 2013). This view has been supported by many empirical studies showing that information is integrated in a nearly Bayes-optimal way in motor control (Wolpert et al., 1995; Ernst and Banks, 2002; Körding and Wolpert, 2004), and also in perceptual decision-making (Yang and Shadlen, 2007; van Bergen et al., 2015).

Körding and Wolpert (2004) showed that previously-learned probability distributions were combined with newly-added probability distributions in a Bayesian fashion, in the performance of a reaching task (Körding and Wolpert, 2004). A cursor indicated the start and end points of the reaching act with a hidden hand, but an experimental shift was added at the end point, using Gaussian noise. In this experiment, human subjects performed the reaching task by directing their index finger to the target. After learning the Gaussian distribution of the experimental shift, (first-probability distribution, i.e., the *prior*), additional feedback was given at the midpoint of the reaching with different Gaussian noises (second probability distribution, the *likelihood*). Subjects’ behavior suggested that the two probability distributions were combined in a nearly Bayes-optimal way.

Brunton et al. (2013) showed that the variability of perceptual decision-making depends on sensory noise but not on the noise in the information-accumulation processes in an auditory pulse number-discrimination task as detailed in a mathematical model (Brunton et al., 2013). Because the information accumulation processes can be regarded as Bayesian inference (Bitzer et al., 2014), their findings suggest that the information accumulation for the perceptual decision-making is nearly Bayes-optimal.

On the other hand, models that assume bias in the information accumulation process, such as in Decision Field Theory and in the “leaky competing accumulator” model (Busemeyer and Townsend, 1993; Usher and McClelland, 2001), have also been proposed to explain the contextual effects (i.e., compromise effect, attractiveness effect, and similarity effect), as well as the primacy/recency effect. These models take into account deviations from Bayesian inference, as explained below. Interestingly, Powers et al. (2017) recently reported that hallucinations, abnormal perception in schizophrenia, can be explained by such deviations.

Classical economic decision-making studies have assumed that humans behave optimally (von Neumann and Morgenstern, 1947). However, the existence of cognitive biases in probability judgment has been amply shown (Kahneman et al., 1982). There is a well-known question that leads people to answer incorrectly: “It is known that the probability of contracting this disease is 1/10,000 and correct rates of positive and negative results obtained by a test for the disease are 99% for both ill and healthy subjects. Now, supposing the test results were positive for you. What would you estimate the probability is that you are actually ill?” People tend to answer greater than the correct rate such as “90%” despite the fact that, (mathematically), the correct answer is about 1% (0.99 ⋅ 0.0001 / (0.99 ⋅ 0.0001 + 0.01 ⋅ 0.9999) ≈ 0.0098). Here, they are considered to ignore the prior (or “base rate neglect”) (Kahneman et al., 1982). Other biases such as “representativeness bias,” “conservatism,” and “anchoring and adjustment” can also be considered as deviations from optimal Bayesian inference (Kahneman et al., 1982).

Thus, various psychological phenomena have been described as optimal Bayesian inference or systematic deviations, pertaining perceptual decision-making and economic decision-making or probability judgment. However, there have been only limited attempts to explain perceptual decision making and economic decision-making within a unified framework (Summerfield and Tsetsos, 2012).

In this paper, we use the perspective of generalized Bayesian inference by considering the bias, and explain various decision-making, probability judgment, and cognitive control. We also consider the possibility that the brain actually implements such biased Bayesian inference.

First, we describe a Bayesian inference model with two exponential biases. Next, we arrange major parameter estimation methods [maximum likelihood (ML) estimation, maximum posterior probability (MAP) estimation and usual Bayesian estimation] in a two-dimensional parameter space of biased Bayesian inference. This parameter space also accounts for biases in probability judgment which have served as a basis for the development of behavioral economics. Furthermore, we discuss the neural implementation of Bayesian inference with exponential biases on the basis of neural connections weight changes (Goldman et al., 2009), regarded as a combination of the leaky competing accumulator model (LCA) (Usher and McClelland, 2001), and probabilistic population codes (PPC) (Ma et al., 2006). Finally, we apply our framework to cognitive control.

## Biased Bayesian Inference

The Bayesian method is a powerful tool that enables inference and decision-making even with a limited amount of data, succeeding where traditional statistical methods have failed to capture inherent dynamics. This method has been applied in many research fields, such as Genetics, Linguistics, Image processing, Cosmology, Ecology, Machine learning, Psychology, and Neuroscience (Stone, 2013).

In all these cases, the solutions were based on a very simple Bayes’s theorem, which states that the probability of the hypothesis H after observation of data D is proportional to the product of the likelihood within the received data, and the prior probability of the hypothesis, or: (*P*(*H*|*D*) ∝ *P*(*D*|*H*) *P*(*H*)). However, when it comes to human and animal behavior, deviations from Bayes-optimality have been observed in many cases (Kahneman et al., 1982). In some studies, such deviations have been explained by introducing exponential biases (i.e., inverse temperature parameters), on Bayesian inference (Nassar et al., 2010; Soltani and Wang, 2010; Payzan-LeNestour and Bossaerts, 2011, Payzan-LeNestour and Bossaerts, 2012; Payzan-LeNestour et al., 2013), mainly because these were found useful in expressing bias levels. Each exponential bias can be separately considered as a bias for a corresponding single distribution, and they are credited for introducing separate biases for prior probability and likelihood.

**FIGURE 1:**
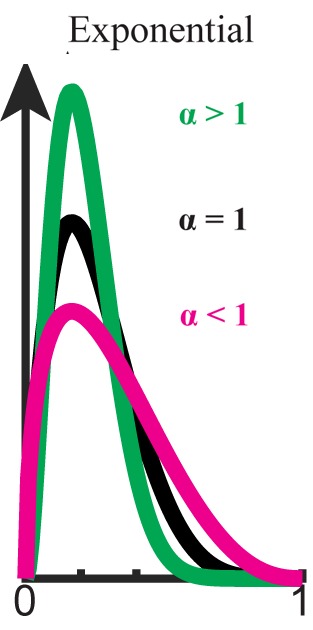
Exponential bias (P ∝ P^α^,0 ≤ α). Beta distribution is used here for graphical demonstration. Original distribution (black) is flattened when α < 1 (magenta) and sharpened when α > 1 (green).

Here, We Propose the Importance of Introducing Both Biases on Prior Probability and Likelihood Simultaneously (see **Figure 1**).

$$P(H|D)\propto P{\left(D|H\right)}^{\beta }P{\left(H\right)}^{\alpha }$$

where ∝ stands for proportionality, α is the exponential bias of the prior probability and β is the exponential bias of the likelihood. Equation (1) can be taken as its logarithm,

log⁡(P(H|D))=β⁢log⁡P(D|H)+α⁢log⁡P(H)+const.

where α is the weight of the logarithm of prior probability, β is the weight of the logarithm of likelihood, and *const.* is the constant term for normalization (see **Supplementary Material**). α and β was regarded as *biases* in Equation (1) but as *weights* in Equation (2). We will take the former term, *biases*, in the present paper so as to keep consistency throughout this paper and emphasize the deviation from the optimal inference. Some authors have expressed Bayes’ theorem as the sum of the log-likelihood ratio and odds ratio (Grether, 1980; Soltani and Wang, 2010). However, they only considered a 2-alternative choice task, which cannot address interesting phenomena such as preference reversals observed in an alternative choice task with more than two choices, while most decisions we make are among more than two choices (Churchland and Ditterich, 2012).

Here, we assume that the bias levels (α,β) are positive (but see **Supplementary Material**). When α = β = 1, the inference is just as in usual Bayesian inference. When the weight of the prior distribution in the inference is smaller than usual (0 < α < 1), the prior distribution is flatter than the original. In this case, the influence of the initial prior on the posterior probability distribution weakens as the prior is updated (see **Supplementary Material**). This type of biasing in inference is called *forgetting*, because the older the information becomes, the less influence it has (Peterka, 1981; Nassar et al., 2010; Payzan-LeNestour and Bossaerts, 2011, Payzan-LeNestour and Bossaerts, 2012). In a non-stationary environment fluctuating gradually, the older information is less important than the newer in predicting the next state. Thus, *forgetting* enables us to take into account the non-stationarity of the environment when we do not have a complete model of the environment (Peterka, 1981; Kulhavý and Zarrop, 1993).

**FIGURE 2:**
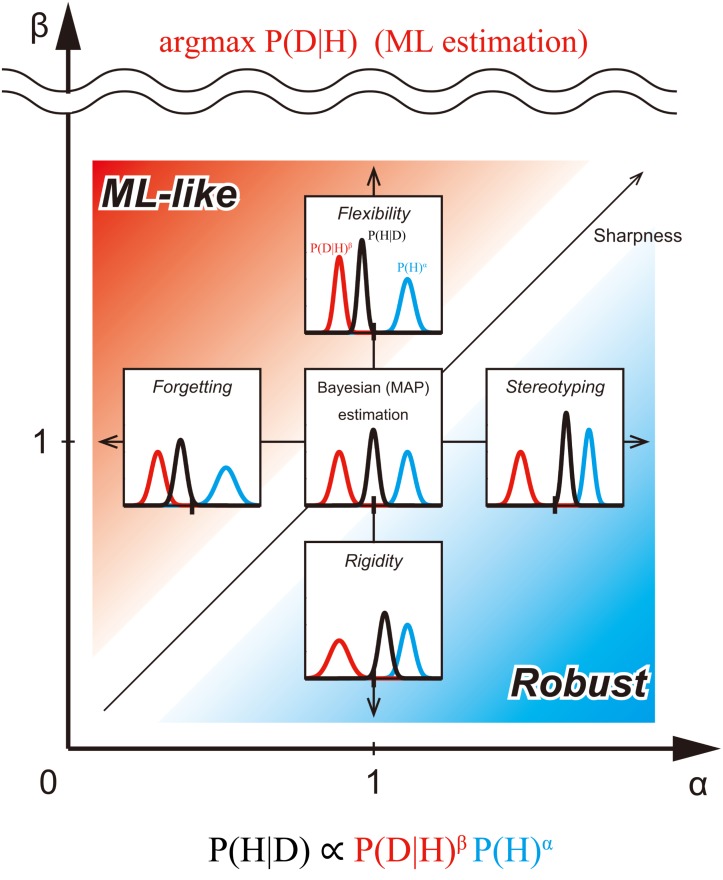
Exponentially biased Bayesian inference (*P*(*H*|*D*) ∝ *P*(*D*|*H*)^β^ *P* (*H*)^α^) in the bias plane. We consider the bias plane provided by the two parameters, the weight of prior distribution (α) and the weight of likelihood (β). Each point in the bias plane corresponds to the different inference methods and provides corresponding posterior distribution from a pair of prior and likelihood (representative values of the distribution-like mode can be taken, but they are not considered here). The inserted graphs show the inferences assuming the same normal distributions at five typical points in the plane. Standard (non-biased) Bayesian inference corresponds to the point with α = β = 1. In the region of 0 ≤ α < 1, the prior distribution is flatter than the original one. In this case, the influence of the initial prior on posterior probability distribution weakens as the prior is updated. This type of biasing in inference is called *forgetting* (Peterka, 1981; Kulhavý and Zarrop, 1993). In the region of α > 1, the prior distribution is sharper than the original. In this case, the influence of the prior on posterior probability distribution is stronger than usual. We call this type of biasing *stereotyping*. In the region of 0 ≤ β < 1, the likelihood is flatter than the original one. In this case, the influence of the likelihood on the posterior probability distribution is weak. We call this type of biasing *rigidity*. In the region of β > 1, the likelihood is sharper than the original one. In this case, the influence of the likelihood on posterior probability distribution is greater. We call this type of biasing *flexibility*. When β approaches +∞, the posterior distribution converges to the mode of the likelihood [the same result as Maximum likelihood (ML) estimation]. Thus, *flexibility* provides an intermediate method between Bayesian and ML estimations. The red region (α < β), emphasizes likelihood more than the prior. We call the inference of this region *ML-like* Bayesian inference. The blue region (α > β), however, emphasizes prior more than likelihood. We call the inference of this region *Robust* Bayesian inference. In the region where α, but not β, is close to 0, the shape of the posterior is mostly determined by likelihood alone. In the region where β (but not α), is close to 0, the shape of the posterior is mostly determined by the prior alone. In the region where both α and β are close to 0, the shape of the posterior is almost flat.

When the weight of the prior distribution in the inference is larger than usual (i.e., α > 1), the prior distribution is sharper than the original (see **Figure 2**). In this case, the influence of the prior on the posterior probability distribution is stronger than usual. We call this type of biasing *stereotyping*, because the influence of old information never decreases, but in fact increases.

When the weight of the likelihood in the inference is smaller than usual (i.e., 0 < β < 1), the likelihood is flatter than the original. In this case, the influence of the likelihood on the posterior probability distribution is weak. We call this type of biasing *rigidity*, because observed data is taken less into account. When the observed data are outliers, the posterior distribution can be greatly influenced by the data. *Rigidity* reduces the influence of such outlier data (Agostinelli and Greco, 2012, 2013).

When the weight of the likelihood in the inference is larger than usual (i.e., β > 1), the likelihood is sharper than before. In this case, the influence of the likelihood on posterior probability distribution is greater. We call this type of biasing *flexibility*, because the observed data is strongly influential on the posterior probability. If β → +∞, the posterior distribution becomes the mode of the likelihood. Thus, *flexibility* allows for a compromise between Bayesian estimation and Maximum Likelihood (ML) estimations (see **Figure 2**).

Here, we would like to inform readers that more than two sources of data can be considered simultaneously. In such cases, the likelihood weights of the data sources (β_1_, β_2_, …) might be different; (see section *Cognitive control as gain modulation and biased Bayesian inference*).

### Parameter Estimation Based on the Biased Bayesian Inference

Standard Bayesian inference has been used for Bayesian estimation and Maximum a-posteriori (MAP) estimation. In the previous section, we introduced Bayesian inference with exponential biases. Next, we consider a variety of parameter estimation methods in the bias plane (α - β plane) for biased Bayesian inference (**Figure 2**).

Each point in the bias plane provides a corresponding posterior distribution based on a pair of priors and likelihood. So, each point corresponds to the different inference methods. We can consider different estimation methods depending on what is returned, as well as representative values of the distribution such as mean, median and mode, or the distribution itself.

In the case of returning the representative value of distribution (here, in this paper, focusing on mode), the point with (α,β) = (1,1) corresponds to the standard MAP estimation. Since the likelihood is weighted more than the prior in the region where α < β is satisfied, (as shown above the 45° line in **Figure 2**), we call the estimation of this region, *ML-like* estimation. In this region, we can consider intermediate estimation between MAP and ML. In particular, when α = *k*_1_ and β = 1, the inference corresponds to the ML estimation if *k*_1_ = 0, MAP estimation if *k*_1_ = 1, and the MAP-ML intermediate estimation (*forgetting*), if 0 < *k*_1_ < 1 (horizontal arrow in **Figure 2**). Furthermore, when α = 1 and *k*_2_ = 1/β, the inference corresponds to the ML method if *k*_2_ = 0, MAP estimation if *k*_2_ = 1, and another type of MAP-ML intermediate estimation (*flexibility*), if 0 < *k*_2_ < 1, (vertical arrow in **Figure 2**).

By contrast, since the prior is weighted more than the likelihood in the region where α > β is satisfied (below the 45° line in **Figure 2**), we call the estimation of this region the “*robust estimation.*”

In this region, we can consider intermediate estimation between MAP (and, so to speak), “*maximum prior (MP)*” estimation. In particular, when α = *k*_1_ and β = 1, the inference corresponds to MAP estimation if *k*_1_ = 1, the *MP* estimation, if *k*_1_ → +∞, and the MAP-*MP* intermediate estimation (*stereotyping*), if *k*_1_ > 1 (horizontal arrow in **Figure 2**). Furthermore, when α = 1 and *k*_2_ = 1/β, the inference corresponds to the MAP estimation if *k*_2_ = 1, the *MP* method if *k*_2_ → +∞ and another type of MAP-*MP* intermediate estimation (*rigidity*), if *k*_2_ > 1; (vertical arrow in **Figure 2**).

In the case of returning distribution itself, the point with (α,β) = (1, 1) corresponds to the standard Bayesian estimation. In the *ML-like* region, when α = 1 and *k*_2_ = 1/β, the inference corresponds to the ML method if *k*_2_ = 0 because it is considered a point estimation that returns arg max *P*(*D*|*H*) (as the sharpness of likelihood should be the maximum above the scale break in **Figure 2**), thus, Bayesian estimation results if *k*_2_ = 1, and the Bayesian-ML intermediate estimation (*flexibility*) when 0 < *k*_2_ < 1 (**Figure 2**). In the *Robust* region, when α = 1 and *k*_2_ = 1/β, the inference corresponds to the Bayesian estimation if *k*_2_ = 1; the inference just returns the prior if *k*_2_ → +∞, and the Bayesian-*prior* intermediate estimation (*rigidity*), if *k*_2_ > 1 (vertical arrow in **Figure 2**).

### Mapping the Psychological Biases of Probability Judgment on the Bias Plane

In this section, we map the psychological biases on the bias plane (**Figure 2**). In the context of human probability judgment, many deviations from Bayesian inference are known, such as base-rate fallacy, representativeness bias, conservation, anchoring and adjustment etc. (Tversky and Kahneman, 1974; Kahneman et al., 1982). In the bias plane, base-rate fallacy and representativeness correspond to the *ML-like* estimation when α < β (as in **Figure 2**), because the likelihood is more influential than the prior in these deviations. Here, we emphasize the distinction of the bias which comes from *forgetting* (α < 1), and from *flexibility* (β > 1), which are similar in the aspect that the posterior distribution is drawn more to the likelihood rather than the prior, but arises from different mechanisms (Brock, 2012; Pellicano and Burr, 2012a; **Figure 2**).

Conservation and anchoring-and-adjusting correspond to *robustness* (where α > β), because the prior is more influential than the likelihood in these deviations. Here we emphasize the distinction of the bias which comes from *stereotyping* (i.e., when α > 1), and from *rigidity* (when β < 1), which are similar in the aspect that the posterior distribution is drawn more from the prior than the likelihood, but comes from different mechanisms (**Figure 2**). Note that our approach is applicable to variable problem situations and ones with more than two alternatives, while most previous attempts to quantitatively represent these biases have considered only two simple alternatives (Grether, 1980; Soltani and Wang, 2010; see also section 2AFC).

Biased Bayesian inference enables us to take into account the non-stationarity of the environment when we do not have a complete model of the environment. So, these psychological biases may have adaptive meanings and normative account as suggested by previous studies (Kulhavý and Zarrop, 1993; Gigerenzer and Goldstein, 1996). Their studies propose that the bias framework presented here provides insights into the sources of influences on these biases.

## Neural Implementation of Biased Bayesian Inference

### Neural Representation of the Probability Distribution

**FIGURE 3:**
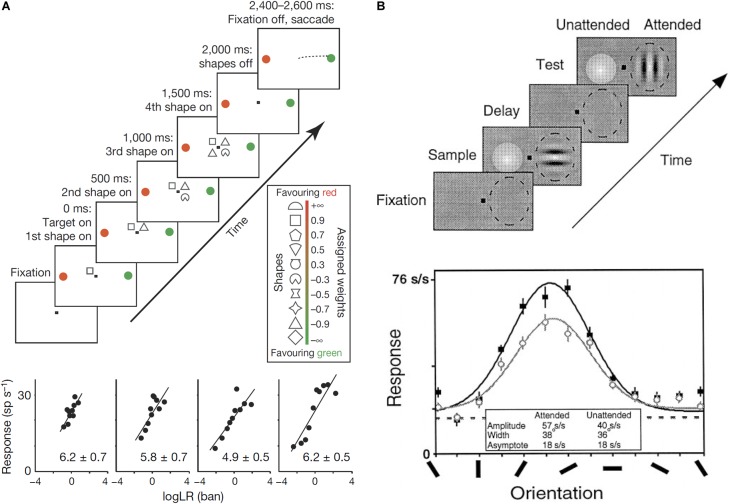
**(A)** Log-probability coding. The task sequence used by Yang and Shadlen (2007) (top). After four shapes were presented sequentially around the central fixation point, the monkey made a saccade to either the red or green choice target. One of the choice targets was in the response field of the recorded neuron. The shapes were selected randomly in each trial from a set of 10 shapes, which are associated with different weights (log likelihood ratio, log LR), for preferred target location (inset). The reward was determined probabilistically by summing the log LR associated with the four shapes. Firing rate is linearly correlated with the summed log LR (bottom). **(B)** Gain modulation by attention. Sequence of the delayed match-to-sample task used by McAdams and Maunsell (1999) (top). While the monkey was gazing at the fixation point and holding a lever, a Gabor stimulus was presented in the receptive field of a recorded neuron (dashed oval) with a colored Gaussian stimulus outside the receptive field during the sampling period. Whereas the monkey was required to pay attention to the orientation of the Gabor pattern in the attended mode, it was required to pay attention to the color of the Gaussian one in the unattended mode. After the delay period with no stimulus except for the fixation point, a Gabor and a Gaussian appeared again and the monkey had to report whether the test stimulus at the attended location matched the sample stimulus during the test period by releasing (match case), or maintaining, hold of the lever. Tuning curves of one V4 neuron for the attended mode (solid symbols) and the unattended mode (open symbols). The response amplitude significantly increased in the attended mode relative to the unattended mode without significant changes in the width or base line activity.

Some authors have proposed computational models in which neural circuits perform Bayesian inference (Jazayeri and Movshon, 2006; Ma et al., 2006). Neural circuits need to represent probability distributions to perform Bayesian inference. Since synaptic inputs usually work additively (Kandel et al., 2000), Bayesian inference can be achieved by summation of neural activity in the brain. To make it possible to achieve Bayesian inference by summation, the neural activity should represent a logarithm of probabilities. In fact, several empirical studies have reported that neurons encode posterior probability in logarithmic form (**Figure 3A**; Yang and Shadlen, 2007; Kira et al., 2015).

Two theoretical methods have been proposed to encode the log probability distribution in a neural population (Pouget et al., 2013; Ma and Jazayeri, 2014). The first method is log probability coding in which the firing rate of each neuron is proportional to the log or logit of the probability of the event or belief coded by the neuron (Barlow, 1969; Jazayeri and Movshon, 2006; Pouget et al., 2013; **Figure 3A**). By simply pooling the activity of the neurons considered, the probability distribution can be represented by the neural population (Yang and Shadlen, 2007; Pouget et al., 2013). The second, more sophisticated method, is probabilistic population coding (PPC), in which a basis function is combined with the log probability coding. And log *P*(*x*) can be written as follows (Ma et al., 2006; Pouget et al., 2013):

$$\log P(x)=\underset{i}{\Sigma }r_{i}h_{i}(x)+const.=r\cdot h+const.$$

where **r***_i_* is the firing rate of ith neuron, **r** = (**r**_1_, **r**_2_, …, **r**_*n*_)*^T^*, *h*__*i*_ (*x*) is basis function for an ith neuron, and **h** = (*h*_1_ (*x*), *h*_2_ (*x*), …, *h_n_* (*x*))*^T^*.

In this paper, we propose a neural model of biased Bayesian inference on the basis of PPC.

### Implementing Biased Bayesian Inference in a Neuronal Population

**FIGURE 4:**
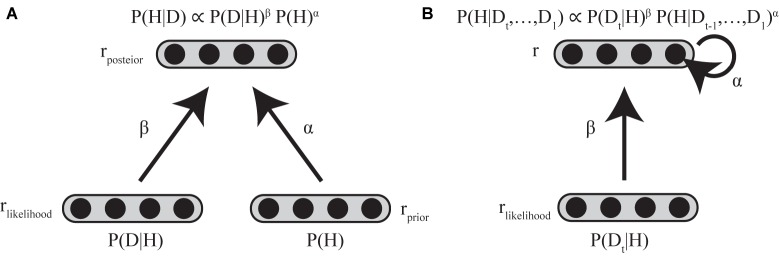
Neural implementation of biased Bayesian inference **(A)** Feed-forward implementation. The first layer consists of two neural populations encoding prior and likelihood, and the second layer consists of neural populations encoding posterior distribution. r_prior_, r_likelihood_, and r_posterior_ indicate the firing rates of the corresponding populations. Standard Bayesian inference is achieved by summing firing rates of corresponding neurons between the neuronal population encoding prior and that encoding likelihood at the next layer of the neuronal population encoding the posterior distribution (*r_posterior_* = *r_prior_* + *r_likelihood_*) in case that the firing rates are proportional to log probabilities (Ma et al., 2006; Yang and Shadlen, 2007). The biased Bayesian inference can be achieved just by changing the gain (α,β) of the inputs for the next (posterior) layer (*r_posterior_* = α*r_prior_* + β*r_likelihood_*). **(B)** Recurrent implementation. The first layer consists of neural population encoding likelihood, and the second layer consists of neural population encoding of prior/posterior distributions. r_likelihood_ and r indicate the firing rates of the corresponding populations. Input to the second layer that encodes prior/posterior distribution is αr + β*r*_likelihood_ (*t*). α is strength of recurrent connection, β is the strength of feed-forward connection.

In considering the implementation of standard and biased Bayesian inference below, we assume two layers of neuronal populations with identical basis functions (see **Supplementary Material**). The first layer consists of two neural populations encoding the prior and the likelihood, and the second layer consists of a neural population encoding the posterior distribution (**Figure 4A**).

In PPC, standard Bayesian inference is achieved by summing the firing rates of corresponding neurons between the neuronal population encoding the prior, and that encoding the likelihood, at the next layer of a neuronal-population-encoded posterior distribution, because summation of their log probabilities is equivalent to the product of the prior and likelihood (Ma et al., 2006).

In this paper, we introduced biased Bayesian inference with exponential biases above (*P*(*H*|*D*) ∝ *P*(*D*|*H*)^β^ *P* (*H*)^α^). Biased Bayesian inference can be achieved just by changing the gain (α,β) of the inputs for the next (posterior) layer considered in the implementation of the standard Bayesian inference (Equation 4; **Figure 4A**).

$$\begin{array}{c} \log\left(P{\left(D|H\right)}^{\beta }P{\left(H\right)}^{\alpha }\right)=\beta \,\log P(D|H)+\alpha \,\log P\left(H\right)\\ =\beta r_{likelihood}\cdot h+\alpha r_{prior}\cdot h+const.\\ =(\beta r_{likelihood}+\alpha r_{prior})\cdot h+constt.\\ =r_{posterior}\cdot h+const. \end{array}$$

where **r***_prior_*, **r***_likelihood_* and **r***_posterior_* are the firing rates of neural populations encoding the prior, likelihood, and posterior, respectively. α and β correspond to the bias for connection weights between prior-encoding neurons and posterior encoding-neurons and the bias for connection weight between likelihood-encoding neurons and posterior-encoding neurons, respectively. Thus, **r***_posterior_* is calculated as β**r***_likelihood_* + α**r***_prior_*. Such gain modulation of neuronal firing rates is ordinarily used in the brain (Desimone and Duncan, 1995; Desimone, 1998) without clearly sophisticated, high cost mechanism, so biasing by gain modulation may be good a strategy of the brain to provide solutions that are approximately optimal.

### Recurrent Connection and Neural Integrator

In Bayesian updating, the posterior distribution generally becomes the prior distribution for the following iterative time step. The resolution of the time steps for Bayesian updating depends on the task; it is trial-based when sensory evidence for updating the prior is given only at the end of a trial (Glimcher, 2003), whereas it is based on finer temporal steps in a single trial when sensory evidence is sequentially (or continuously) given throughout the trial (Bogacz et al., 2006; Kira et al., 2015). Such repetitive updating can be achieved regardless of the temporal resolution by a recurrent neural circuit, in which the firing rates of neurons last across the time steps (Goldman et al., 2009; Bitzer et al., 2014; Kira et al., 2015; Chandrasekaran, 2017). Neural integrator models have been considered as a mechanism for maintain the firing rate with external and recurrent inputs (Goldman et al., 2009). Since the dynamics of neural integrators can be described using a firing rate equation (Dayan and Abbott, 2001; Goldman et al., 2009), biased Bayesian inference can therefore be expressed by Equation (5) (**Figure 4B**).

$$\tau_{neuron}\frac{dr}{dt}=-r+Input=-r+\alpha r+\beta r_{likelihood}\left(t\right)$$

where τ*_neuron_* is the intrinsic decay time constant of neurons, **r** represents the firing rate of the neurons that encode prior/posterior distribution, and Input is input to the neurons that encode prior/posterior distribution (α**r** + β**r***_likelihood_* (*t*)), α is strength of recurrent connection, β is strength of feedforward connection and **r***_likelihood_* is the firing rate of the neuronal population that encodes the likelihood (with external input to be integrated).

In the neural integrator model, the strength of recurrent input required for neurons to independently maintain their firing rates is α (Goldman et al., 2009). When a neuron maintains its firing rate in the absence of external input, the neural integrator is called *balanced* (α = 1), because the firing rate changes due to recurrent input and intrinsic decay are regarded as balanced. If the firing rate decreases due to weak recurrent input, it does not perfectly compensate for the intrinsic decay of the firing rate, and therefore, the neural integrator is called *leaky* (α < 1), and in this case, encoded distribution decays over time (*forgetting*). If the firing rate increases due to recurrent input and overcompensates for the intrinsic decay of the firing rate, the neural integrator is called *unstable* (α > 1). In this case, encoded distribution is sharpened over time (what we call *stereotyping*).

### Linking Biased Bayesian Inference to the Two-Alternative Forced Choice (2AFC)

2AFC decision-making has been well-studied (Stone, 1960; Ratcliff, 1978; Busemeyer and Townsend, 1993; Gold and Shadlen, 2001; Roe et al., 2001; Shadlen and Newsome, 2001; Usher and McClelland, 2001, 2004; Wang, 2002; Mazurek et al., 2003; Bogacz et al., 2006; Wong and Wang, 2006; Kiani et al., 2008; Tsetsos et al., 2012), because this is one of the simplest cases of decision-making.

The accumulator models of 2AFC such as the Drift-diffusion model (DDM) (Ratcliff, 1978), decision field theory (DFT) (Busemeyer and Townsend, 1993; Roe et al., 2001), and the leaky competing accumulator model (LCA) (Usher and McClelland, 2001, 2004), typically make three assumptions:

(i) Evidence favoring each alternative is integrated over time in a single trial;
(ii) The process is subject to random fluctuations; and,
(iii) A decision is made when sufficient evidence has accumulated favoring one alternative over another (Bogacz et al., 2006).

These models are drawn from neuronal data particularly in monkey lateral intraparietal cortex (LIP) (Kiani et al., 2008; Brunton et al., 2013).

DDM uses an optimal integrator, and this model can be seen as the continuum limit of the Sequential Probability Ratio test (SPRT) – the optimal sequential hypothesis-testing method (Neyman and Pearson, 1933; Wald and Wolfowitz, 1948; Wald, 1973; Bogacz et al., 2006; Kira et al., 2015). Because the instantaneous drift is considered the likelihood, the instantaneous drift of DDM can be considered as a form of Bayesian inference (Bogacz et al., 2006; Bitzer et al., 2014) by taking the starting point of accumulation as the initial prior.

**FIGURE 5:**
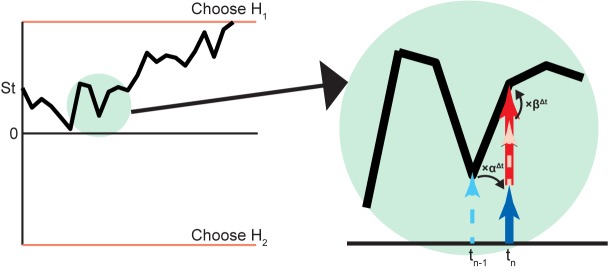
Biased Bayesian inference in diffusion model of 2AFC. An example of the decision process used to choose between two hypotheses (H_1_,H_2_) on the basis of the diffusion model is illustrated on the left. The vertical axis is the log posterior probability ratio of hypotheses ($\log\frac{P\left(H_{1}|Data\right)}{P\left(H_{2}|Data\right)}$) and the horizontal axis is time. The diffusion starts from $St=\log\frac{EV\left(H_{1}\right)}{EV\left(H_{2}\right)}=\log\frac{P(H_{1})\cdot {Rwd}_{1}}{P(H_{2})\cdot {Rwd}_{2}}$ and develops until it reaches either top or bottom threshold (orange line) for decision. (The diffusion obviously starts from 0 when *P* (*H*_1_) ⋅ *Rwd*_1_ = *P* (*H*_2_) ⋅ *Rwd*_2_). The part in the green circle is magnified to show detail (right). The log posterior probability ratio at t_n-1_ (dashed cyan arrow) becomes log prior probability ratio at t_n_ (solid blue arrow) with the bias α^Δt^, where Δt = t_n_ - t_n-1_. In this case, the solid blue arrow is shorter than the dashed cyan arrow, indicating that α < 1 (*forgetting*). This can be implemented by a leaky integrator (**Figure 4B**) [Usher and McClelland, 2001]. At t_n_, the original log likelihood ratio (dashed pink arrow) inputs as the biased (×β^Δt^) log likelihood ratio (solid red arrow), and the log posterior probability ratio is made by adding the biased log likelihood ratio to the log prior probability ratio. In this case, the solid red arrow is longer than the dashed pink arrow, indicating β > 1 (*flexibility*).

In contrast to DDM, DFT, and LCA assume leaky neural integrators instead of the optimal integrator, thereby explaining many behavioral biases such as the primacy/recency effect and three preference reversal effects (compromise effect, attractiveness effect, and similarity effect) (Busemeyer and Townsend, 1993; Roe et al., 2001; Usher and McClelland, 2001, 2004; Bogacz et al., 2006) in two- or multi-alternative forced choice tasks. However, the normative Bayesian perspective has never been applied to DFT or LCA. A simplified form of the DFT or LCA, the Ornstein–Uhlenbeck process (Bogacz et al., 2006), is consistent with the recurrent network model (**Figure 4B**), whose inputs to the second layer have Gaussian noise. Thus, DFT and LCA can be considered forms of biased Bayesian inference (**Figure 5**). Here, we would like to emphasize that biased Bayesian inference is a key tool in combining the descriptive richness of models of 2AFC and the normative Bayesian perspective which are implemented in PPC (Beck et al., 2012) (**Supplementary Material**).

In addition to Bayesian inference, an intrinsic gain/loss function should also be considered in the selection of “an action in the environment” (Ernst and Bulthoff, 2004). When different reward values are contingent on corresponding action selections, the reward function *Rwd* (*x*) dependent on an executed action x must also be considered (Matsumoto et al., 2003, 2006; Matsumoto and Tanaka, 2004). The *Rwd* (*x*) can be encoded by population firing rates *r_reward_*, as posterior probability *P* (*x*) denoted here as *r_prior/posterior_*. Since the expected value of reward *EV* (*x*) is calculated by multiplying the probability of obtaining the reward with the reward value on the basis of action *x*, the combination between the Bayesian inference and intrinsic gain/loss function may be obtained by simple multiplication of *Rwd* (*x*) and *P* (*x*). In other words, *EV* (*x*) can be denoted as *r_prior/posterior_* + *r_reward_*, where, (log *EV* (*x*) = log *P* (*x*) + log *Rwd* (*x*)), (in considering that the firing rates are proportional to log probabilities). Actually, recent studies have shown that the difference in reward between directions changes the starting point of integration, which corresponds to the prior knowledge, irrespective of mean drift rate in the diffusion model on the basis of fronto-parietal activity (Rorie et al., 2010; Summerfield and Koechlin, 2010; Mulder et al., 2012). Thus, Bayesian inference and the calculation of the expected value of reward can be achieved by the same mechanism if one takes the reward value as a prior (Friston et al., 2013).

Importantly, the mean drift rate in value integration is also modulated by spatial visual attention (Krajbich et al., 2010). This finding suggests that the level of exponential bias for *Rwd* (*x*) changes depending on attention, which could be shifted more by the higher value of reward associated with the target processed in the fronto-striatal network (Schultz, 2006; Hikosaka, 2007).

### Empirically Testable Predictions in 2AFC Tasks

There is some concern that Bayesian approaches in psychology and neuroscience are often “just-so stories” because it can often provide many degrees of freedom for explanations and they are not falsifiable (Bowers and Davis, 2012). In the comment paper for Bowers and Davis (2012) and Griffiths et al. (2012) wrote: “In evaluating claims about falsifiability, it is useful to distinguish between a model and a theoretical framework. A model is proposed to account for a specific phenomenon and makes specific assumptions in order to do so. A theoretical framework provides a general perspective and a set of tools for making models. […] Models are falsifiable, but frameworks are typically not” (Griffiths et al., 2012).

Here, we provide some falsifiable predictions about the behavioral and neural models of biased Bayesian inference for 2AFC tasks, which is a typical model on the basis of our biased Bayesian inference, the theoretical framework in the present paper.

As for the biased Bayesian inference behavioral models for 2AFC task, it would be easily falsified merely if the decision processes were not based on the prior, likelihood, and gain/loss function, because our model for 2AFC assumes the subjects’ decision processes are based on them as typical Bayesian inference model is. “Bayesian transfer” is a useful experimental procedure to determine whether a decision process is based on the prior, likelihood, and gain/loss function (Maloney and Mamassian, 2009). In this procedure, subjects are overtrained for two decision tasks which contain different priors, likelihoods, and gain/loss functions (Task 1, Task 2) until subjects’ performances come close to maximizing expected gain in both tasks. Then, it is tested whether the subjects can transfer the knowledge about priors, likelihoods, and gain/loss functions acquired in Task 1 and Task 2 into a new task (transfer task). For example, the transfer task contains the same likelihood and gain/loss function as those in Task 1 and the same prior as that in Task 2. The subjects are familiar with the prior, likelihood, and gain/loss function in the resulting transfer task, but the combination of prior, likelihood, and gain function is novel. One can state that subjects’ decision processes are based on the prior, likelihood, and gain/loss function when the subjects’ performances in the transfer task immediately close to ideal without further practice or learning (Maloney and Mamassian, 2009). Actually, Bayesian transfer should be efficiently applied to 2AFC tasks, because the prior and gain/loss function can be easily changed by instruction and the likelihood functions is easily switched with another in the tasks.

Even if the biased Bayesian inference behavioral models for 2AFC passed the Bayesian transfer test, the models would be further falsified if they were non-biased. This falsification check is important because it has been considered that the information accumulation for the perceptual decision-making is nearly Bayes-optimal rather than biased or leaky/unstable, at least in short time integration (Gold and Shadlen, 2007; Brunton et al., 2013). In order to check whether the integration is leaky/unstable (α≠1) by behavioral data with the assumption of constant α and β, one can use psychophysical reverse correlation (Ahumada, 1996; Neri et al., 1999; Okazawa et al., 2018). Psychophysical reverse correlation is a technique that estimates how sensory information is weighted to guide decisions by quantifying the spatiotemporal stimulus fluctuations that precede each choice (Okazawa et al., 2018). If sensory weights are constant throughout the integration process, the integration can be seen as perfect integration. Otherwise, one can state that the integration is leaky/unstable.

As for the biased Bayesian inference neural models, we proposed it on the basis of the empirical findings that LIP neurons encode the accumulated information (prior/posterior distribution) and middle temporal cortex (MT) neurons encode instant sensory information (likelihood function) (Mazurek et al., 2003; Gold and Shadlen, 2007). Therefore, the biased Bayesian inference neural models would be falsified, simply if firing rates of LIP neurons were explained by perfect integration (α = 1) because it would just deny our assumption of biased, leaky/unstable integration (α≠1).

The other bias for likelihood (β) can also be considered. One may be interested in the biases originated from inference process itself (β*_connection_*), which is different from the biases originated from representation of probability distributions (β*_rate_*) (**Supplementary Material**). One can state that the origin of bias is inference process itself (β*_connection_*≠1) if the choice behaviors change associated with the corresponding changes in firing rates of LIP neurons without changes in firing rate of MT neurons. Actually, such a situation can be available in switching between two tasks, one to discriminate motion direction and the other to discriminate stereoscopic depth in the same moving random dot stereogram stimuli (Sasaki and Uka, 2009; Kumano et al., 2016). Therefore, by using such a context-dependent task switching, the biased Bayesian inference neural models whose biases originated from inference process itself would be falsified, if firing rates of LIP and MT neurons were rather consistent to the neural models with the biases originated from representation of probability distributions.

## Cognitive Control as Gain Modulation and Biased Bayesian Inference

We have already seen that the biased Bayesian inference can be explained by gain modulation in neuronal populations. Here, we explain how the bias levels may be regulated by cognitive control.

Cognitive control is thought to occur in two steps: first, the anterior cingulate cortex (ACC) monitors cognitive control demands on the basis of uncertainty in response to outcomes and sends signals to the prefrontal cortex (PFC), and then, the PFC recruits this cognitive control by sending top–down signals to various cortical areas (Botvinick et al., 2001, 2004; Matsumoto, 2004; Shenhav et al., 2013).

### Application to Stroop task

The Stroop task is a well-known task that has been used to examine the neural mechanisms of cognitive control. This task consists of word reading and color naming. In the word reading task, the subject is asked to report the written color name of a color, printed in the same color (*congruent*); or another (*incongruent*) (wherein, “green” is the answer for GREEN printed in red). Since human subjects in modern countries are trained more to read words as opposed to reporting colors, relatively low cognitive control is recruited in this task. In the color naming task, a subject is asked to name the ink color of a word denoting the same color (*congruent*) or a different color (*incongruent*) (e.g., “red” being the correct answer for the word “GREEN” printed in red), and relatively high cognitive control must be recruited to overcome the cognitive interference. Particularly, the reaction times are longer and the error rates are higher in the *incongruent* condition than in the *congruent* one – what is now known as the “Stroop effect” (Stroop, 1935).

**FIGURE 6:**
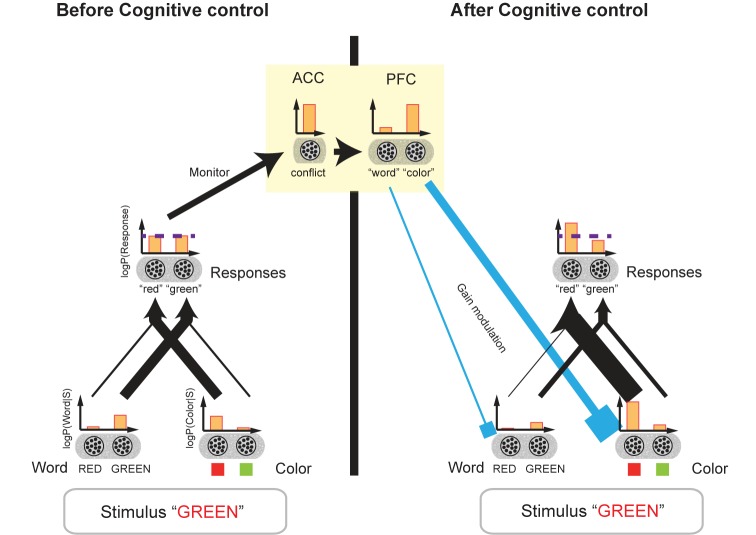
Biased Bayesian inference and cognitive control. The network model of cognitive control for the Stroop task (Botvinick et al., 2001) is shown. This model can be considered a network model of biased Bayesian inference. The bar graphs indicate the mean firing rates of the neural populations, and their outputs to the target populations is indicated by the connecting arrows’ thickness which is consistent with the firing rates. Before cognitive control, when the stimulus [“GREEN” in red ink color ((*incongruent*) in this example)] is presented, word-encoding neurons and color-encoding ones in the perception layer are activated (*r*(*RED*) < *r*(*GREEN*), *r*(*green link*); *r*(●) being the mean firing rate of the population). We assume that these neurons encode the log-likelihoods of the word and color of the presented stimulus. The perception layer sends output to the response layer. The *incongruent* stimulus, “GREEN” printed in red, activates not only the neurons encoding “green” but also the neurons encoding “red” in the response layer. Then, the firing rates of the two types of neurons should be close to each other *r*(“*red*”) = *r*(“*green*”); the horizontal dashed line indicates the original level of the firing rates]. (If the congruent stimulus, “GREEN” printed in green, for example, were presented, the firing rates of the two types of neurons should be distinctively different from each other.) We assume the neurons in the response layer encode the prior/posterior distribution of the responses. The comparable firing rates of the neurons encoding different responses are considered a conflict, which thus activates the ACC. The ACC then sends the cognitive control demand signal to the PFC (yellow square). In turn, the PFC regulates the gains of word- and color-encoding neurons by sending a top–down signal (cyan input), which corresponds to regulation of bias levels (β*_word_*,β*_color_*) in our biased Bayesian inference. The top–down signal augments the gains of neurons encoding task-relevant information (color encoding neurons in this example), and then the firing rates of the two types of neurons in the response layer (“green,” “red”) begin to differ in accordance with the task requirements. Thus, the appropriate response is achieved.

Botvinick and colleagues proposed a neural network model of cognitive control focusing on the conflict monitoring function of the ACC (**Figure 6**; Botvinick et al., 2001, 2004; Shenhav et al., 2013). Here, we show that this model can be considered a neural model of a Bayesian network with biases (Rao, 2005). When the stimulus is presented, word-and color-encoding neurons in the perception layer are activated. We conjecture that these neurons encode log-likelihoods of word and color.

The succeeding response layer receives input from the perception layer. We conjecture that the neurons in the response layer encode prior/posterior distributions for responses such as “green,” “red” and so on; (here, a flat prior being assumed). According to Botvinick’s model, because we are more adept at reading words rather than naming colors due to the more prevalent experience of enforcement on reading, the connection between the word-encoding neurons and the response layer is stronger than that between the color-encoding neurons and the response layer.

An *incongruent* stimulus, however (e.g., “GREEN” printed in red), activates not only the neurons encoding “green” but also the neurons encoding “red” in the response layer. And, accordingly, the responses of the two types of neurons should be closer to each other than the responses to the congruent stimulus, i.e., “GREEN” printed in green. The comparable firing rates of the neurons encoding different responses come into conflict, thus activating the ACC. The ACC is then believed to send a cognitive control signal to PFC. In turn, the PFC regulates the gains of word and color encoding neurons through a top–down signal, which amounts to regulation of bias levels (β*_word_*, β*_color_*) in our biased Bayesian inference (**Figure 6**). The top–down signal augments the gains of neurons encoding task-relevant information (i.e., word-encoding neurons in the word reading task, and color-encoding neurons in the color naming task), and then the firing rates of the two types of neurons in response layers for “green” and “red” become differentiated in accordance with task requirements. The result being, that the appropriate response is achieved.

### Application to Working Memory

Cognitive control works in a variety of situations requiring flexible behaviors that use goal-directed, top–down selection of relevant information, as in, “maintain it tentatively despite irrelevant distractors, and utilize it to solve a problem” (a process akin to working memory) (Baddeley, 1986).

Now, we consider working memory within the framework of biased Bayesian inference. Top–down attentional regulation of working memory can be regarded as being governed by neural integrators whose gains are considered the biases in a biased Bayesian inference.

Working memory is actively maintained longer than several seconds even across distracting stimuli (Miller et al., 1996; Miyake and Shah, 1999). Persistent activity based on top–down attention might be sustained by a reciprocal positive feedback loop within a population of neurons in certain cortical regions including the PFC (Curtis and D’Esposito, 2003; Curtis and Lee, 2010).

The attentional top–down signals from the PFC improve working memory (Desimone and Duncan, 1995; Miller and Cohen, 2001; Noudoost et al., 2010; Gazzaley and Nobre, 2012), suggesting that the top–down signals work to change the gain of neural connections implemented in working memory circuits. Here, the attentional top–down signals should contribute to adaptive modulation of either α or β (Norman and Shallice, 1986; Desimone and Duncan, 1995; McAdams and Maunsell, 1999; Botvinick et al., 2001; Miller and Cohen, 2001; Egner and Hirsch, 2005; Maunsell and Treue, 2006; Reynolds and Heeger, 2009; Cole et al., 2013, 2014), as the gain of the neural connections corresponds to α in the biased Bayesian inference. Therefore, this can be regarded as a change in the balance of neural integrators (i.e., α = 1 corresponds to the balanced integrator, α < 1 to the leaky integrator, and α > 1, to the unstable integrator; Roe et al., 2001; Gazzaley and Nobre, 2012).

### Application to Top–Down Attention

Top–down attention amplifies the tuning curves of activity in sensory cortical regions (McAdams and Maunsell, 1999; Treue and Trujillo, 1999; Maunsell and Treue, 2006; **Figure 3B**). This is because the tuning curve amplification itself can be considered a form of noise reduction (Dayan and Zemel, 1999), and the effect of the bias (β) on the target of attention should be based on the top–down attentional signal that reduces the noise of the target.

Tuning curves are not completely flattened even for task-irrelevant features such as distractors. For example, it is well known that subjects’ behaviors are affected by distractors in a variety of tasks that require top–down attention (Stroop, 1935; Eriksen and Eriksen, 1974). This cannot be explained by standard, non-biased Bayesian inference assuming binary βs for task-relevant and task-irrelevant features, but by biased Bayesian inference that can take arbitrary values as for the βs. This can be considered a Bayesian version of the biased competition model (Desimone and Duncan, 1995; Desimone, 1998).

Here, we do not make strong assumptions of normality for represented probability distributions as Friston et al. (2006) and Friston (2009), and Friston (2010) did in their considerations of gain modulation in the context of predictive coding based on a free energy principle. Our approach sacrifices the mathematical tractability of theirs, and instead makes the models applicable to recently developed decision-making and cognitive control theories by allowing the representation of arbitrary probability distributions.

One important issue is how to determine the bias levels as suggested by Shenhav et al. (2013). Also, we would like to note to readers that some studies have treated the prior as attention (Angela and Dayan, 2004; Chikkerur et al., 2010). However, these issues are beyond the scope of this paper.

## Possible Relationship Among Neuromodulators/Neurotransmitters, Gain Modulation, and Psychiatry

Detailed mechanisms of gain modulation and application to psychiatry are also important (**Supplementary Material**) (Friston et al., 2006; Friston, 2009; Friston, 2010; Mante et al., 2013; Miller and Buschman, 2013), since psychiatric diseases could be caused by changes in connectivity between the PFC and other regions which lead to defects in cognitive control (Cole et al., 2014; Stephan et al., 2015).

Potent candidates that modulate gain for cognitive or top–down attention control are classical neuromodulators/neurotransmitters such as norepinephrine, acetylcholine, glutamate, λ-aminobutyric acid (Friston et al., 2006; Friston, 2009; Friston, 2010).

### Norepinephrine

**FIGURE 7:**
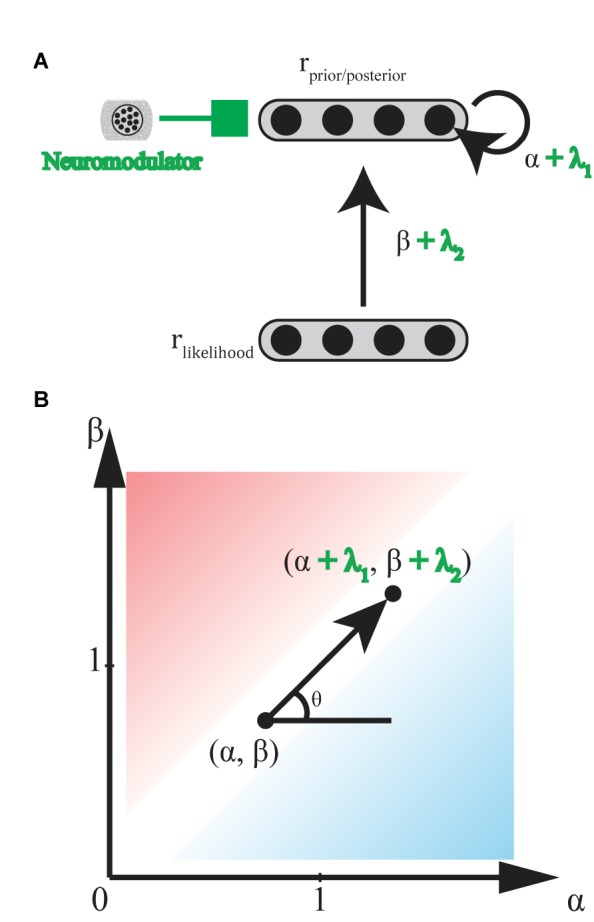
Hypothetical effect of neuromodulators/neurotransmitters. **(A)** General scheme of neural implementation of biased Bayesian inference with a neuromodulators/neurotransmitter. The first layer consists of neural population encoding likelihood, and the second layer consists of neural population encoding prior/posterior distribution. α + λ_1_ is the strength of the recurrent connection, andβ + λ_2_ is strength of the feedforward connection. λ_1_ and λ_2_ are the effects of neuromodulators/neurotransmitters on recurrent and feedforward connections, respectively. **(B)** Hypothetical effect of neuromodulators/neurotransmitters on bias plane. Neuromodulators/neurotransmitters may change the bias levels in the inference from (α,β) to (α + λ_1_, β + λ_2_) where λ_1_, λ_2_ ≥ 0 (0° ≤ θ ≤ 90°) for norepinephrine, and λ_1_ ≤ 0, λ_2_ ≥ 0 (90° ≤ θ ≤ 180°) for acetylcholine. Excitatory/Inhibitory (E/I) balance of neurotransmitters, glutamate and GABA, might also be regarded as variations in the bias levels in inference. Whereas λ_1_, λ_2_ ≥ 0 (0° ≤ θ ≤ 90°) when E is greater than I, and λ_1_, λ_2_ ≤ 0 (180° ≤ θ ≤ 270°) when I is greater than E.

Norepinephrine (NE)-containing cells in the locus coeruleus (LC) receive ongoing task utility values [as evaluated by ACC and orbitofrontal cortex (OFC)], release NE at cortical areas involved in stimulus judgment and behavioral decision making to modulate the gain of current task-relevant information specifically (exploitation) or possible alternatives (exploration) (Servan-Schreiber et al., 1990; Aston-Jones and Cohen, 2005; Yu and Dayan, 2005). This gain modulation corresponds to bias levels (α and β) in the biased Bayesian inference, where the sharper distribution is led by higher levels of NE (**Figure 7**).

Attention deficit/hyperactivity disorder (ADHD) patients show NE dysfunction. They not only show erratic trial-to-trial exploratory behavior but also high trial-to-trial variability in their reaction times in 2AFC tasks with probabilistic reinforcement (Frank et al., 2007; Hauser et al., 2016). Consistent with these behavioral findings, a computational simulation suggests that appropriate NE release leads to sharper motor cortical representations and sharper distribution of reaction times (Frank et al., 2007).

### Acetylcholine

Acetylcholine (ACh) release from basal forebrain cells modulates the gain of processing in cortical circuits involved in memory in two ways (Hasselmo and McGaughy, 2004; Hasselmo, 2006). ACh enhances the cortical responses to afferents inputting new inputs for encoding on one hand, but suppresses the cortical responses to recurrent feedback for memory maintenance on the other hand. Thus, whereas high ACh levels enhance new encoding, low ACh levels enhance maintenance and following consolidation. Yu and Dayan (2005) proposed a consistent model suggesting that ACh levels are high when a presented stimulus does not indicate an appropriate response with certainty, (here, called the expected uncertainty), while other inputs may still be informative. This gain modulation corresponds to a function with an ML-like-robust axis (β - α) in the biased Bayesian inference (**Figure 7**): where high and low ACh levels correspond to ML-like and robust inferences, respectively (but see Hasselmo, 2006; Hasselmo and Sarter, 2011).

Patients with Alzheimer’s disease (associated with ACh hypofunction), show working memory impairment (Baddeley et al., 1991). This may be caused by robust inference on the basis of a least input (reduced β), and/or *unstable* neural integrator (enlarged α).

### Excitatory/Inhibitory (E/I) Balance

Glutamate (Glu) and gamma-aminobutyric acid (GABA), respectively, act as important excitatory and inhibitory neurotransmitters in the brain (Kandel et al., 2000). The ratio of these two neurotransmitters (i.e., “E/I balance” in a functional unit of neural circuitry), should determine the gain of intrinsic activity in the circuit. This gain corresponds to α in the biased Bayesian inference. The functional unit of neural circuitry receives plenty of glutamatergic and GABAergic inputs externally. Thus, the E/I balance of these external inputs should determine the gain of the activity which they evoke in the circuit. This gain corresponds to α and β in the biased Bayesian inference (**Figure 7**).

The E/I balance in cerebral cortex is altered in schizophrenic patients (Wang, 2001; Kehrer et al., 2008; Murray et al., 2014). There are two major positive symptoms of schizophrenia: hallucinations (i.e., perceiving something that is not actually there), and delusions (erroneous beliefs that usually involve a misinterpretation of perceptions or experiences). It has been suggested that neither hallucinations nor delusions occur when gain levels based on both prior knowledge and external input from the environment are correct. On the basis of the predictive coding hypothesis, Corlett et al. (2009) have suggested that hallucinations occur when the gain level of prior knowledge exceeds that of external input and delusions occur when the gain level of external input exceeds that of prior knowledge (Corlett et al., 2009; Fletcher and Frith, 2009; Teufel et al., 2015; Powers et al., 2017). Because the gain levels of prior knowledge and external input are expected to depend on the E/I balance of their corresponding connections, hallucinations and delusions can be explained by an abnormal E/I balance.

Autism is a heritable, lifelong neurodevelopmental condition characterized by difficulties in social communication and social interaction (called social symptoms), and a range of restricted activities and sensory abnormalities (called non-social symptoms) (Pellicano and Burr, 2012b). Some forms of autism are also thought to be caused by an altered E/I balance in sensory, mnemonic, social and emotional systems (Rubenstein and Merzenich, 2003; Nelson and Valakh, 2015). Since E/I balance determines bias levels in the biased Bayesian inference, the altered E/I balance in autism implies inappropriately-biased neural processing (**Figure 4**). Here, we focus on the process causing a sensory abnormality in autism, since the construction of a Bayesian model for social communication and social interaction has not yet reached consensus.

As we normally experience strong visual illusions such as in the Kanizsa triangle (Kanizsa, 1955) and Shepard’s table (Shepard, 1990), the brain postulates the most likely interpretation for the noisy, ambiguous sensory signals corresponding to strong priors (Pellicano and Burr, 2012b). By contrast, individuals with autism are less susceptible to such illusions and show more accurate or overly realistic perceptions, suggesting that their priors are flattened (Pellicano and Burr, 2012b) (i.e.,α < 1, or *forgetting* in the bias plane). However, as Brock (2012) has noted, the same results can be obtained with a sharpened likelihood, when β > 1 (with *flexibility* in the bias plane) as well, because these two biases make similar posterior distributions (ML-like inferences), as we have explained in **Figure 2**.

## Limitations

The biased Bayesian models especially in the context of 2AFC tasks would be empirically identifiable to some extent as described in section Empirically Testable Predictions in 2AFC Tasks. However, details of neural implementation of biased Bayesian inference such as discrimination between the biases originated from inference process and those originated from representation of probability distributions and importance of gain/loss function in the model identifiability issue are not fully addressed. Also, the proposed models for cognitive control and those based on assumed neuromodulators/neurotransmitters functions for understanding mental disorders are insufficient to test their identifiability. Because the identifiability issue should be severer due to more parameters than that for usual Bayesian models, rigorous model comparisons must be done in future studies that apply the biased Bayesian models.

## Conclusion

In the present paper, we have discussed the importance of introducing exponentially-biased Bayesian inference into a cognitive framework with testable neural correlates. The bias plane for the biased Bayesian inference is useful in providing a view-integrating ML estimation, Bayesian estimation, and MAP estimation, thus enabling us to make intermediate parameter estimations between ML estimation and Bayesian (or MAP) estimation. It also explains many psychological decision biases, such as base-rate fallacy, representativeness, conservation, anchoring-and-adjusting, primacy/recency effect, three-preference reversal effects (compromise effect, attractiveness effect, and similarity effect), as well as cognitive control. Furthermore, it may provide further insight into the mechanisms of ADHD, Alzheimer’s disease, and schizophrenia (including hallucinations and delusions), and, potentially, autism as well.

## Author Contributions

KaM conceptualized the data. KaM and KeM wrote the paper. KaM, KeM, and YK revised the paper.

## Conflict of Interest Statement

The authors declare that the research was conducted in the absence of any commercial or financial relationships that could be construed as a potential conflict of interest.
