# Supplementary material for: A Biased Bayesian Inference for Decision-Making and Cognitive Control
Source: Front Neurosci. 2018 Oct 12;12:734. doi: 10.3389/fnins.2018.00734 (PMC6195105; doi:10.3389/fnins.2018.00734)
Supplement: Supplementary file 1 [file Data_Sheet_1.pdf]

frontiers

Supplementary Material

A Biased Bayesian Inference for Decision-Making and Cognitive Control

Kaosu Matsumori, Yasuharu Koike, Kenji Matsumoto*

*** Correspondence:** Dr. Kenji Matsumoto: matsumot@lab.tamagawa.ac.jp

1. Properties of biased Bayesian inference

1.1 The exponential family and biased Bayesian inference

The exponential family (EF) is very important in Bayesian statistics because if probability distributions belong to an EF, they have *sufficient statistics* and *conjugate priors* as well as other convenient properties. Here we consider relations between EF and exponential biases. When a probability distribution $P(x)$ belongs to EF, $P(x)^{\alpha}$ also belongs to the EF. And, if the *base measure* of the distribution does not depend on x, raising it to the power of α corresponds to multiplying it by the *natural parameter* by α. This makes the calculation much easier. In the case that the distribution is a normal distribution, multiplying the *natural parameter* by β is equivalent to multiplying the variance by $1/\beta$. So, biased Bayesian inference can be seen as a Bayesian version of the weighted least squares method.

1.2 Exponential and linear type biases

One idea for modifying probability distributions is to combine probability distributions. A combined distribution needs to be close to both of the previous distributions. Kullback–Leibler (KL) divergence can be used to measure the distance between probability distributions. Kulhavý and Kraus (1996) considered two methods of combination by minimizing KL divergence [1]. This is expressed as

$$p = \arg\min_{P}\left[(1-\alpha)KL\left(P\|P_1\right)+\alpha KL\left(P\|P_0\right)\right] \quad (1)$$
$$p = \arg\min_{P}\left[(1-\alpha)KL\left(P_1\|P\right)+\alpha KL\left(P_0\|P\right)\right] \quad (2)$$

where P_0 and P_1 are probability distributions. P is the combined distribution. Note that KL divergence is asymmetric, such that ($KL\left(P_0\|P_1\right)\neq KL\left(P_1\|P_0\right)$). The combined distribution P is close to both P_0 and P_1. If P_0 is prior and P_1 has a uniform distribution, Equation 1 corresponds to exponential biases ($P \propto P_0^{\alpha}P_1^{1-\alpha} \propto P_0^{\alpha}$) (Fig. 1) and Equation 2 corresponds to linear biases ($P = \alpha P_0 + (1-\alpha)P_1$) (Fig. S1) [1,2]. In this paper, we have focused on exponential biases because exponential bias can make the original distribution either flatter or sharper, whereas the linear bias can only make it flatter and cannot be considered when the probability distribution is defined for infinite domains (but see Supplementary Material *4 Details of neural implementation of biased Bayesian inference*).

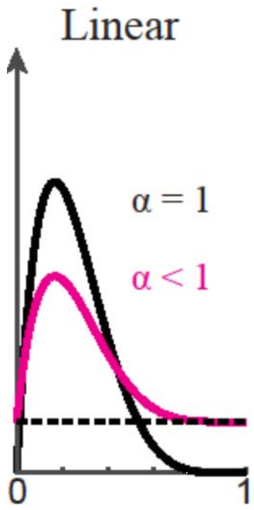

Figure S1. Linear bias ($P = \alpha P + (1-\alpha)\cdot Uniform$, $0 \le \alpha \le 1$). Beta distribution is used here for graphical demonstration. The original distribution (black) is flattened when $\alpha < 1$ (magenta). The dashed line is the uniform distribution component of the modified distribution. Exponential bias can make the original distribution not only flatter but also sharper, whereas linear bias only makes the original distribution flatter.

2 *Non-positive bias levels and exponential bias as standard Bayesian inference*

In the main text, we only assumed that bias levels (α, β) are positive, because posterior distribution cannot always be normalized when the bias levels are non-positive.

When β is 0, it is an ordinary case and the posterior distribution is just the same as the prior. However, when α is 0, this is a special case in which the prior becomes a uniform distribution on an infinite interval. Although the biased prior is not a probability distribution since it cannot be normalized, the posterior can be normalized if the likelihood function can be normalized. In this case, MAP estimation and ML estimation give the same results.

When α and/or β are negative, these biases generate more or less the inverse of the original, non-biased distribution of prior and/or the likelihood. (Such an inverse might be implemented by inhibitory synapses in the brain (see Supplementary Material 4 Details of neural implementation of biased Bayesian inference)). The size-weight illusion, which has been considered as apparently "anti-Bayesian", can actually be explained by negative α [3,4].

Moreover, once we allowed the bias levels to be negative, exponential biases for probability distributions could be expressed by standard Bayesian inference. By assuming the special case in which the likelihood $P(D|H)$ matches the power function of prior ($P(H)^{\beta}$) with $\beta > -1$, an exponentially-biased prior ($P(H)^{1+\beta}$) can be generated by standard Bayesian inference, with ($P(H)^{1+\beta} \propto P(D|H)P(H) = P(H)^{\beta} P(H)$). Therefore, our exponentially-biased Bayesian inference can also be considered a form of hierarchical standard Bayesian inference.

3 *Forgetting*

3.1 Repeated Biased Bayesian inference

After n updates, the posterior distribution of biased Bayesian inference is:

$$\log P\left(H \mid D_n, ..., D_1\right) = \sum_{t=1}^{n}\left\{\left(\prod_{i=t+1}^{n}\alpha_i\right)\beta_t \log P\left(D_t \mid H\right)\right\} + \left(\prod_{i=1}^{n}\alpha_i\right)\log P\left(H\right) + const. \tag{3}$$

where n is the number of updates, D_t is t^{th} data and the initial prior is $P\left(H\right)$. So, the influence of the initial prior is diminishes by inference if $\alpha_t < 1$.

This enables the posterior to have a certain upper limit of sharpness, even if the distribution is updated by same likelihood function infinite times.

3.2 Exponential smoothing and biased Bayesian inference

Exponential smoothing is one of the techniques that have been broadly applied to smooth time-series data, without intending to be strictly accurate or reliable for every situation.

Exponential smoothing is:

$$\mu_{t+1} = wd_t + \left(1 - w\right)\mu_t$$

where μ_t is the estimated value at t, d_t is the obtained data at t, and w is the weight for obtained data. Exponential smoothing is also used in reinforcement learning, such as when $w = 0.1$ [5]:

$$\mu_{t+1} = \mu_t + w\left(d_t - \mu_t\right) = \mu_t + w\delta$$

Here, prediction error $\delta = d_t - \mu_t$. The learning rate w is a free parameter. It is important to properly determine w. An appropriate learning rate can be automatically determined by the standard Bayesian method. We assume that the prior for variable S and that the likelihood for data D is normal such that

$$P\left(S\right) \sim N\left(\mu_t, \sigma_t\right), P\left(D \mid S\right) \sim N\left(\mu_S, \sigma_S\right),$$

and the posterior distribution is:

$$P\left(S \mid D\right) \sim N\left(\tfrac{\sigma_t^2}{\sigma_t^2 + \sigma_S^2}\mu_S + \tfrac{\sigma_S^2}{\sigma_t^2 + \sigma_S^2}\mu_t, \tfrac{\sigma_t^2\sigma_S^2}{\sigma_t^2 + \sigma_S^2}\right).$$

Thus,

$$\mu_{t+1} = w_t\mu_S + \left(1 - w_t\right)\mu_t = \mu_t + w_t\left(\mu_S - \mu_t\right) = \mu_t + w_t\delta\ ,$$

and

$$\sigma_{t+1} = \sqrt{\tfrac{\sigma_t^2\sigma_S^2}{\sigma_t^2 + \sigma_S^2}}$$

where $w_t = \sigma_t^{\ 2} / \sigma_t^{\ 2} + \sigma_S^{\ 2}$. In this case, the learning rate w_t becomes smaller and smaller. Here, usual exponential smoothing ($w = w_1, w_2, ...$) can be interpreted as biased Bayesian inference. If $\alpha = 1 - w_t$, so $\sigma_{t+1} = \sigma_t$. Thus, $w = w_1, w_2, ...$. In this case, biased Bayesian inference (forgetting)

is the same as exponential smoothing. Therefore, biased Bayesian inference in Cognitive Sciences is justified because it is equivalent to exponential smoothing or using a fixed learning rate in reinforcement learning.

4 *Details of neural implementation of biased Bayesian inference*

4.1 Biases on strength of connection and firing rates

In the main text, we considered only the case that the first layer of Fig. 4a encodes the correct distribution. In that case, it was enough to consider the biases on the strength of connection (α, β in the main text). However, the firing rate of the population of the second layer is affected by changes in the firing rate of the first layer populations as well as in the strength of connection between the first and second layers, which are based on biologically distinct mechanisms. Therefore, we can also consider the case that the firing rates of first layers themselves are already biased by changing the firing rate gain parameters $(\alpha_{rate}, \beta_{rate})$ in addition to the biases on the strengths of connection. When we assume that $\mathbf{r}_{prior}$ and $\mathbf{r}_{likelihood}$ are the firing rates of the two populations in the first layer of Fig. 4a that encode correct distributions for prior ($P(H)$) and likelihood ($P(D|H)$), the biased firing rates of these populations should be $\alpha_{rate}\mathbf{r}_{prior}$ and $\beta_{rate}\mathbf{r}_{likelihood}$, and the distributions encoded by these biased firing rates should be $P(H)^{\alpha_{rate}}$ and $P(D|H)^{\beta_{rate}}$. Here, we denote the biases on the strength of connection as $\alpha_{connection}$ and $\beta_{connection}$ (instead of α and β) separately from the firing rate gain parameters. Thus, the firing rate of the second layer is $\alpha_{connection}\alpha_{rate}\mathbf{r}_{prior} + \beta_{connection}\beta_{rate}\mathbf{r}_{likelihood}$. We introduce α_{total} and β_{total} as a product of the biases on the strength of connection and changing firing rate gain parameters as ($\alpha_{total} = \alpha_{connection}\alpha_{rate}$, $\beta_{total} = \beta_{connection}\beta_{rate}$). The discussion above is also true for the case of recurrent networks (Fig. 4b).

Because it is not necessary to discriminate between α_{total} and α or between β_{total} and β in considering how the models work, we did not discriminate between them in the main text.

4.2 Additional lateral interactions between neurons

In the main text, we considered only the case that all biases on neurons in the same population have the same value (α or β). This enables biased Bayesian inference if the neuronal populations encoding prior, likelihood and posteriors have a common basis function which does not assume any lateral interaction. However, if the basis functions of the neuronal populations are different, we need to introduce connection matrices ($\mathbf{A}$, $\mathbf{B}$), not scalars (α, β), for the biases, in which lateral interactions are considered [6]. Let a_{ij} and b_{ij} be matrix $\mathbf{A}$ and $\mathbf{B}$'s $(i\ ,j)^{th}$ entries. a_{ij} corresponds to the strength of the connection between the jth neuron in the prior layer and i^{th} neuron in the posterior layer. b_{ij} corresponds to the strength of the connection between the jth neuron in the likelihood layer and the ith neuron in the posterior layer.

In turn, α and β are equivalent to the products between themselves and the identity matrix $\mathbf{I}$ ($\alpha\mathbf{I}$ and $\beta\mathbf{I}$). In considering the recurrent network (Fig. 4b), we can add interactions between

neurons in the second layer by putting non-zero values in non-diagonal elements of the connection matrix. (Actually, LCA and DFT introduced lateral inhibition by modulating the elements including non-diagonal ones [7,8,9]).

It may also be important to consider distance-dependent lateral inhibition when choice options are represented in a certain parameter space [10], but this goes beyond the scope of this paper because modulation of elements depends on the shape of the tuning function and is not part of our current study.

4.3 Linear bias approximated by exponential bias

When the current state S_t stays at probability α with respect to the previous and shifts with probability $1-\alpha$ to another state that is sampled from $P\left(S_{shift}\right)$ in the next, the linear combination of the probability distributions which correspond to stay and shift ($\alpha P\left(S_t\right)+\left(1-\alpha\right)P\left(S_{shift}\right)$) becomes the next prior in the Bayesian sense. This is considered a kind of linear bias in *1 Properties of biased Bayesian inference*. Yu and Cohen (2008) showed that the linear combination is approximately achieved by using a leaky integrator [11]. In our view, this means that the linear bias can be approximated by the exponential bias in such cases.

References

1. Kulhavý R, Kraus F (1996) On duality of regularized exponential and linear forgetting. Automatica 32: 1403-1415.

2. Kulhavý R, Zarrop MB (1993) On a general concept of forgetting. International Journal of Control 58: 905-924.

3. Peters MA, Ma WJ, Shams L (2016) The Size-Weight Illusion is not anti-Bayesian after all: a unifying Bayesian account. PeerJ 4: e2124.

4. Brayanov JB, Smith MA (2010) Bayesian and "anti-Bayesian" biases in sensory integration for action and perception in the size-weight illusion. J Neurophysiol 103: 1518-1531.

5. Sutton RS, Barto AG (1998) Reinforcement learning: An introduction.

6. Ma WJ, Beck JM, Latham PE, Pouget A (2006) Bayesian inference with probabilistic population codes. Nature Neuroscience 9: 1432-1438.

7. Usher M, McClelland JL (2001) The time course of perceptual choice: the leaky, competing accumulator model. Psychological review 108: 550.

8. Busemeyer JR, Townsend JT (1993) Decision field theory: a dynamic-cognitive approach to decision making in an uncertain environment. Psychological review 100: 432.

9. Roe RM, Busemeyer JR, Townsend JT (2001) Multialternative decision field theory: A dynamic connectionst model of decision making. Psychological review 108: 370.

10. Furman M, Wang XJ (2008) Similarity effect and optimal control of multiple-choice decision making. Neuron 60: 1153-1168.

11. Yu AJ, Cohen JD. Sequential effects: superstition or rational behavior?; 2009. pp. 1873-1880.
